# The effect of depressive symptoms on pain in a substance-using population with persistent pain: a cross-sectional cohort study

**DOI:** 10.1186/s12888-021-03424-7

**Published:** 2021-08-20

**Authors:** Pauline Voon, Jin Cheol Choi, Kanna Hayashi, M-J Milloy, Jane Buxton, Thomas Kerr

**Affiliations:** 1 British Columbia Centre on Substance Use, 400-1045 Howe Street, Vancouver, BC V6Z 2A9 Canada; 2 School of Population and Public Health, University of British Columbia, 5804 Fairview Avenue, Vancouver, BC V6T 1Z3 Canada; 3 Faculty of Health Sciences, Simon Fraser University, 8888 University Drive, Burnaby, BC V5A 1S6 Canada; 4 Department of Medicine, University of British Columbia, 400-1045 Howe Street, Vancouver, BC V6Z 2A9 Canada

**Keywords:** Depression, Pain, Overdose, Substance use, Heroin, Mental illness

## Abstract

**Background:**

In light of the ongoing opioid overdose crisis, there is an urgent need for research on the impacts of mental health among people presenting with concurrent pain and substance use. This study examined the effect of depressive symptoms on pain severity and functional interference among people who use drugs (PWUD) during a community-wide overdose crisis.

**Methods:**

From December 1st 2016 to December 31st 2018, 288 participants in two cohort studies of PWUD in Vancouver, Canada completed interviewer-administered questionnaires that included the Brief Pain Inventory and PROMIS Emotional Distress–Depression instruments. Generalized linear regression modelling (GLM) was used to examine the cross-sectional effect of depressive symptoms and other confounding factors on pain severity and interference.

**Results:**

Moderate to severe depressive symptoms were significantly associated with greater pain-related functional interference (adjusted β = 1.24, 95% confidence interval [CI] = 0.33–2.15), but not significantly associated with greater average pain severity (adjusted β = 0.22, 95% CI = − 0.3 – 0.82), when controlling for confounding variables. Reported daily heroin use (adjusted β = 1.26, 95% CI = 0.47–2.05) and non-fatal overdose (adjusted β = 1.02, 95% CI = 0.08–1.96) were also significantly associated with greater pain-related functional interference.

**Conclusions:**

In a substance-using population, greater pain-related functional interference was positively associated with depressive symptoms as well as overdose and daily heroin use. These findings emphasize the need to address the functional impact of pain, mental health comorbidity, and high-risk substance use that may contribute to overdose and other harms.

## Background

In recent years, increasing rates of opioid misuse and overdose have reached unprecedented levels, with opioid-related overdose now becoming a leading cause of injury-related death in several countries including the United States, Canada, Australia, Scotland, and Russia [1–4]. In light of the ongoing opioid crisis, there is an urgent need for research that identifies risk factors in populations that may be susceptible to high-risk opioid use and overdose, such as individuals with persistent pain. To this end, mental illness has been identified as a determinant of problematic opioid use and opioid overdose [5], and therefore remains an important area requiring further investigation.

Mental health comorbidity is common among people experiencing pain and among people who use drugs (PWUD), and is linked to a variety of adverse outcomes. Past research has found a higher prevalence of mental health disorders in specialized pain settings [6] and mental health comorbidity has been found to be associated with higher risk of problematic opioid use [7–10]. Other studies have found an increased risk of substance use, including greater risk of opioid misuse, among individuals with chronic pain and mood disorders, particularly among those with major depression [11–13]. Longitudinal studies of chronic pain patients have similarly found that depression significantly increases risk of problematic opioid use, opioid craving, and poor opioid treatment outcomes related to analgesia and adverse effects [7]. Conversely, the pain experience itself may contribute to depressive symptoms through pathways of emotional distress, lower quality of life, or greater psychosocial burden [14–16].

This study aims to add to the literature on concurrent depression, pain, and substance use by examining the relationship that depressive symptoms may have with pain severity and functional interference in PWUD. As well, this study aims to explore the effects of specific substance use factors—such as heroin use, prescription opioid use, cannabis use, and non-fatal overdose—as potentially statistically significant confounders in the relationship between depressive symptoms and pain, which have not been well described in this context. It is hypothesized that depressive symptoms will be significantly associated with greater pain severity, functional interference, and substance use related behaviours and harms.

## Methods

### Participants

The Vancouver Injection Drug Users Study (VIDUS) and the AIDS Care Cohort to evaluate Exposure to Survival Services (ACCESS) are two cohort studies of PWUD that have been described in detail elsewhere [17]. Briefly, participants are adults aged 18 years or older who have either injected illicit drugs in the month prior to enrolment and are HIV-seronegative (VIDUS) or used an illicit drug other than or in addition to cannabis in the month prior to enrolment and are HIV-seropositive (ACCESS). Participants are recruited in Vancouver, Canada through community-based methods including snowball sampling, street outreach, and self-referral; provide written informed consent; complete interviewer-administered questionnaires at baseline and semi-annually; and receive a $40 (CDN) stipend at each study visit. The study instruments administered for both cohorts are identical, with the exception of additional HIV-specific assessments for the ACCESS cohort, and follow-up periods and procedures are harmonized to facilitate combined data analysis and interpretation between the two cohorts as they operate in tandem. Additionally, any VIDUS participants who seroconvert to become HIV-positive after enrolling in the study are merged into the ACCESS cohort. This study used data from eligible interviews completed between December 1, 2016 and December 31, 2018, during which time the participant’s main survey questionnaire and supplemental posttraumatic stress questionnaire must have been completed within 14 days of each other. The study was restricted to participants who reported major or persistent pain within the 6 months prior to their interview, as ascertained by a response of “Yes” to the initial screening question of the Brief Pain Inventory: “Throughout our lives, most of us have had pain from time to time (such as minor headaches, sprains, and toothaches). Have you had pain other than these everyday kinds of pain?”

### Measures

The Brief Pain Inventory – Short Form (BPI-SF) was used to measure past-week average pain severity, measured using a zero-to-ten scale, and average past 24-h functional pain interference, measured using the average zero-to-ten composite score across six functional domains of general activity, mood, walking, relations with other people, sleep, and enjoyment of life. The BPI is a valid and reliable self-reported pain instrument that has been widely used in studies measuring clinical pain intensity and pain interference among substance-using populations [18–21].

The Adult Patient-Reported Outcomes Measurement Information System (PROMIS) Emotional Distress – Depression Short Form [22] was used to measure depressive symptoms: as per the instrument scoring protocol [23], total raw scores were converted into T-scores, interpreted as “Moderate – Severe” depression for T-scores ≥60.0, and “None – Mild” depression for T-scores ≤59.9. Other mental health variables were measured using the Adult PROMIS Emotional Distress – Anxiety Short Form [22, 24], which compared “Moderate – Severe” anxiety (T-score ≥ 60.0) against “None – Mild” anxiety (T-score ≤ 59.9), and the Posttraumatic Stress Disorder (PTSD) Checklist for DSM-5 (PCL-5), which compared a provisional PTSD diagnosis of “Yes” (PCL-5 score ≥ 31) against a provisional PTSD diagnosis of “No” (PCL-5 score < 31) [25, 26]. Given that the PCL-5 was a supplemental questionnaire administered once per participant only, this study employed a cross-sectional analysis of variables obtained from participants’ PCL-5 responses as well as the corresponding main survey questionnaire that was completed most recently (within 14 days) from the time of the PCL-5 questionnaire.

Other potentially confounding variables were identified based on conceptual and demonstrated associations with depression and pain. For instance, several studies have found significant associations between pain and depressive symptoms and various demographic characteristics such as age, sex, and race [11, 12, 27–29]; various substance use characteristics and disorders [11, 12, 27–29]; overdose [30, 31]; and factors related to ability or inability to access health care including addiction treatment [32, 33]. Therefore, the following covariates were considered for this analysis: age (≥ versus < median); sex at birth (male versus female); race (white versus Black, Indigenous, or Person of Color [BIPOC] or other); highest level of education completed (≥ versus < high school); homelessness (yes to staying outside or in tents or in temporary short-term shelters, versus no); incarceration (yes versus no); non-fatal overdose (yes to the question: “In the last 6 months, have you overdosed by accident (i.e., where you had a negative reaction from using too much drugs)?” versus no); binge drug use (yes versus no); injection drug use (yes versus no); inability to access addiction treatment (yes versus no); experiencing barriers to accessing health services (yes versus no); and currently being on prescribed pain medications at the time of interview (yes versus no).

Additionally, the following substance use variables were considered: heroin use (at least daily use versus less than daily use); stimulant use (i.e., cocaine, crack cocaine, or crystal methamphetamine; at least daily use versus less than daily use); non-medical prescription opioid use (at least daily use versus less than daily use); heavy alcohol use (greater than versus less than or equal to four drinks per day or 14 drinks per week for men, or greater than versus less than or equal to three drinks per day or seven drinks per week for women); and cannabis use (at least daily use versus less than daily use). Participants were asked: “In the last six months, which of the following drugs did you use, and how often did you use them?” Participants were then read out a list of substances by the interviewer. For each substance, the frequency of use for that substance was recorded as either: less than once per month, one to three times per month, about once per week, two or three times per week, or at least daily. For any substance used at least daily, the average number of uses per day was recorded for each substance. For each applicable substance, participants were asked about their frequency of injection and non-injection use separately. For prescription opioids, as per the U.S. National Survey on Drug Use and Health [34], participants were asked: “In the last six months, which of the following prescription opiates did you use when they were not prescribed for you or that you took only for the experience or feeling they caused, and how often did you use them?” A chart of prescription opioid drug names and pictures was then shown and read out to the participant, including the following prescription opiates: OxyNEO, OxyContin, Percocet (Percodan, other oxycodone), Tylenol 3 (codeine), morphine (MS Contin, Avinza, Kadian, M-Eslon), Dilaudid (hydromorphone hydrochloride), Demerol (Darvon, meperidine, propoxyphene), Methadone / Methadose (Dolophine), Suboxone, Fentanyl (Durgesic, Actiq), Hydrocodone (Vicodin), Talwin (pentazocine), or Other, in which participants were able to report any other opiate.

Unless otherwise noted, these variables pertain to activities or events that occurred within the 6 months prior to the participant’s interview. Observations with missing responses to these measures were excluded via listwise deletion contingent upon a non-significant (*p* ≥ 0.05) result from Little’s missing completely at random (MCAR) test [35], given that listwise deletion does not produce bias in estimation if missing data is MCAR [36]. All variables were included in Little’s MCAR test as follows: participant identification number, follow-up number, interview date, average pain severity, average pain interference, PTSD, depressive symptoms, anxiety, age, sex, race, highest education completed, homelessness, incarceration, daily heroin use, daily stimulant use, daily non-medical prescription opioid use, heavy alcohol use, daily cannabis use, overdose, binge drug use, injection drug use, inability to access addiction treatment, barriers to accessing healthcare, currently on pain medication, and cohort (ACCESS versus VIDUS).

### Statistical analyses

First, descriptive statistics of participants stratified by depressive symptoms were examined using Mann-Whitney U tests for the continuous zero-inflated pain severity and interference variables, and Pearson’s chi-squared tests and Fisher’s exact tests (for cell counts ≤5) for categorical variables. Second, the data was checked to ensure that the assumptions were met for satisfying the generalized linear model (GLM) procedure (e.g., homoscedasticity, normality of residuals, Shapiro-Wilk normality test). After confirming normal error distributions, bivariate GLMs with identity link functions were fit to estimate the effect of depressive symptoms and their potentially confounding covariates on pain severity and pain interference. Third, multivariate GLMs were fit to estimate the adjusted effect of depressive symptoms on average pain severity and average pain interference using an a-priori-defined confounding model building approach similar to that of Maldonado and Greenland [37] and several other prior studies [38–40]: beginning with the variables found to have *p* < 0.1 from the bivariate analyses, a reduced model was built by removing the covariate that changed the coefficient of the main predictor variable the least when that covariate was removed. This iterative construction of reduced models, with one covariate removed sequentially, was repeated until a final reduced model was arrived at, in which all covariates in the final model would have changed the estimate of the main predictor variable by at least 5 %. This change-in-estimate criterion filters out non-influential variables, such that the remaining covariates in the final reduced model are considered confounding variables to the primary predictor variable. Variance inflation factors (VIFs) were calculated on the predictor variables in the multivariate confounding models in order to diagnose multicollinearity. A sensitivity analysis was performed following the above model building approach but excluding the covariates of PTSD and anxiety, given their potential collinear relationship with depressive symptoms. For all analyses, *p*-values were two-sided and significant associations were defined as *p* < 0.05. All analyses were conducted using R version 4.0.0 (R Foundation for Statistical Computing, Vienna, Austria, 2020).

## Results

At the start of the study period, the total number of enrolled participants expected to participate in follow-up interviews, excluding those who had deceased or withdrew from the study, was 1961. From December 1, 2016 to December 31, 2018, 1377 PWUD completed at least one study visit (i.e., 584 participants lost to follow-up during the study period). Of those, 903 participants reported major or persistent pain; 979 completed the supplemental PCL-5 questionnaire; and 957 had a valid PCL-5 score, leaving 336 participants who reported pain and completed both the main survey questionnaire and the PCL-5 supplemental questionnaire within 14 days of each other. Of these, 48 participants had missing data for either the outcome variable or covariates of interest (missing response rate = 14.3%). Little’s MCAR test did not produce a significant result (*p* = 0.273); therefore, it was determined that the missing data was MCAR and listwise deletion of the missing data was employed.

A remaining total of 288 participants were eligible for inclusion in this analysis. In this sample, 26% of participants reported moderate to severe depressive symptoms. As shown in Table 1, the sample was 66% male, 45% white, with a median age of 51 years (interquartile range [IQR]: 44–57 years). The median average pain severity was not significantly different between participants reporting moderate to severe depressive symptoms (median: 6, IQR: 5–7) compared to participants reporting no to mild depressive symptoms (median: 6, IQR: 4–7, *p* = 0.218). For average pain interference, scores were significantly higher for participants reporting moderate to severe depressive symptoms (median: 6, IQR: 4–7) compared to participants reporting no to mild depressive symptoms (median: 4, IQR: 1–6, *p* < 0.001). Compared to participants reporting no to mild depressive symptoms, participants with moderate to severe depressive symptoms demonstrated significantly higher unadjusted odds of PTSD, moderate to severe anxiety, younger age, ≥ daily stimulant use, non-fatal overdose, binge drug use, and injection drug use, and lower unadjusted odds of taking prescribed pain medication (Table 1).

**Table 1:** Characteristics of 288 people who use drugs, stratified by PROMIS Emotional Distress – Depression score

Characteristic	Moderate – Severe Depressive Symptoms^**a**^ ***n*** (%) ***n*** = 76 (26.4%)	No – Mild Depressive Symptoms^**a**^ ***n*** (%) ***n*** = 212 (73.6%)	Odds Ratio (95% CI)	***p -*** value
**Average pain severity**^a^				
Median (IQR)	6 (5–7)	6 (4–7)	1.07 (0.95–1.21)	0.218
**Average pain interference**^b^				
Median (IQR)	6 (4–7)	4 (1–6)	1.19 (1.08–1.31)	< 0.001
**PTSD**^c^				
Yes	53 (69.7)	75 (35.4)	4.21 (2.39–7.40)	< 0.001
No	23 (30.3)	137 (64.6)		
**Anxiety**^a^				
Moderate – Severe	56 (73.7)	34 (16.0)	14.66 (7.82–27.49)	< 0.001
None – Mild	20 (26.3)	178 (84.0)		
**Age**				
≥ Median	29 (38.2)	114 (53.8)	0.53 (0.31–0.91)	0.028
< Median	47 (61.8)	98 (46.2)		
**Sex**				
Male	46 (60.5)	144 (67.9)	0.72 (0.42–1.25)	0.305
Female	30 (39.5)	68 (32.1)		
**Race**				
White	35 (46.0)	96 (45.3)	1.03 (0.61–1.75)	1.000
BIPOC or other	41 (54.0)	116 (54.7)		
**Highest education completed**				
≥ High school	32 (42.1)	112 (52.8)	0.65 (0.38–1.10)	0.141
< High school	44 (57.9)	100 (47.2)		
**Homeless**^d^				
Yes	15 (19.7)	23 (10.8)	2.02 (0.99–4.12)	0.077
No	61 (80.3)	189 (89.2)		
**Incarcerated**^d^				
Yes	6 (7.9)	6 (2.8)	2.94 (0.92–9.42)	0.118
No	70 (92.1)	206 (97.2)		
**≥ Daily heroin use**^d^				
Yes	23 (30.3)	40 (18.9)	1.87 (1.03–3.39)	0.057
No	53 (69.7)	172 (81.1)		
**≥ Daily stimulant use**^d^				
Yes	42 (55.3)	75 (35.4)	2.26 (1.33–3.84)	0.004
No	34 (44.7)	137 (64.6)		
**≥ Daily non-medical prescription opioid use**^d^				
Yes	3 (4.0)	4 (1.9)	2.14 (0.47–9.78)	0.385
No	73 (96.0)	208 (98.1)		
**≥ Daily cannabis use**^d^				
Yes	21 (27.6)	51 (24.1)	1.21 (0.67–2.18)	0.643
No	55 (72.4)	161 (75.9)		
**Heavy alcohol use**^d^				
Yes	15 (19.7)	22 (10.4)	2.12 (1.04–4.35)	0.058
No	61 (80.3)	190 (89.6)		
**Overdose**^d^				
Yes	17 (22.4)	24 (11.3)	2.26 (1.14–4.49)	0.030
No	59 (77.6)	188 (88.7)		
**Binge drug use**^d^				
Yes	37 (48.7)	55 (25.9)	2.71 (1.57–4.67)	< 0.001
No	39 (51.3)	157 (74.1)		
**Injection drug use**^d^				
Yes	56 (73.7)	121 (57.1)	2.11 (1.18–3.76)	0.016
No	20 (26.3)	91 (42.9)		
**Inability to access addiction treatment**^d^				
Yes	4 (5.3)	3 (1.4)	3.87 (0.85–17.71)	0.082
No	72 (94.7)	209 (98.6)		
**Barriers to accessing healthcare**^d^				
Yes	19 (25.0)	32 (15.1)	1.88 (0.99–3.56)	0.077
No	57 (75.0)	180 (84.9)		
**Currently on pain medication**				
Yes	27 (35.5)	117 (55.2)	0.45 (0.26–0.77)	0.005
No	49 (64.5)	95 (44.8)		

^a^ Denotes activities/events within the week prior to the participant’s interview

^b^ Denotes activities/events within the 24 h prior to the participant’s interview

^c^ Denotes activities/events within the month prior to the participant’s interview

^d^ Denotes activities/events within the 6 months prior to the participant’s interview

Table 2 presents the results of the bivariate and multivariate GLMs examining the effect of depressive symptoms on average pain severity. Moderate to severe depressive symptoms were not found to be significantly associated with average pain severity in the unadjusted (β = 0.36, 95% CI: − 0.24 - 0.95, *p* = 0.245) or adjusted (β: 0.22, 95% CI: − 0.38 - 0.82, *p* = 0.479) models. ≥ Daily stimulant use was significantly associated with higher average pain severity scores in the unadjusted model, but was no longer statistically significant in the adjusted model. Other substance use related covariates (e.g., heroin use, non-medical prescription opioid use, cannabis use, overdose, injection drug use) were not found to be significantly associated with average pain severity. Sex was the only covariate that was statistically significant in both the unadjusted and adjusted models, with male sex being significantly associated with lower average pain severity scores (unadjusted β = − 0.72, 95% CI: (− 1.27 - -0.17, *p* = 0.010; adjusted β = − 0.67, 95% CI: − 1.22 - -0.12, *p* = 0.019).

**Table 2:** Bivariate and multivariate generalized linear regression of the effect of depressive symptoms on average pain severity^a^ (*n* = 288)

	Unadjusted		Adjusted	
Characteristic	β (95% CI)	***p -*** value	β (95% CI)	***p -*** value
**Depressive symptoms**^a^ (Moderate-Severe vs. Mild-None)	0.36 (− 0.24–0.95)	0.245	0.22 (− 0.38–0.82)	0.479
**PTSD**^**b**^ (Yes vs. No)	0.01 (− 0.52–0.55)	0.959		
**Anxiety**^a^ (Moderate-Severe vs. Mild-None)	0.41 (− 0.15–0.98)	0.155		
**Age** (≥ Median vs. < Median)	− 0.30 (− 0.83–0.22)	0.258		
**Sex** (Male vs. Female)	− 0.72 (− 1.27 - -0.17)	0.010	− 0.67 (− 1.22 - -0.12)	0.019
**Race** (White vs. BIPOC or other)	− 0.49 (− 1.01–0.04)	0.071		
**Highest education completed** (≥ vs. < High school)	− 0.35 (− 0.88–0.17)	0.188		
**Homeless**^**c**^ (Yes vs. No)	0.59 (− 0.19–1.37)	0.138		
**Incarcerated**^**c**^ (Yes vs. No)	0.16 (−1.16–1.48)	0.809		
**≥ Daily heroin use**^**c**^ (Yes vs. No)	0.46 (− 0.18–1.10)	0.158		
**≥ Daily stimulant use**^c^ (Yes vs. No)	0.54 (0.01–1.07)	0.048	0.45 (− 0.09–0.99)	0.107
**≥ Daily non-medical prescription opioid use**^c^ (Yes vs. No)	1.29 (− 0.41–3.00)	0.138		
**≥ Daily cannabis use**^c^ (Yes vs. No)	0.17 (− 0.44–0.78)	0.582		
**Heavy alcohol use**^**c**^ (Yes vs. No)	0.16 (− 0.63–0.94)	0.698		
**Overdose**^**c**^ (Yes vs. No)	0.36 (− 0.39–1.11)	0.350		
**Binge drug use**^**c**^ (Yes vs. No)	0.29 (−0.27–0.86)	0.310		
**Injection drug use**^**c**^ (Yes vs. No)	0.20 (−0.34–0.75)	0.461		
**Inability to access addiction treatment**^c^ (Yes vs. No)	0.56 (−1.15–2.28)	0.520		
**Barriers to accessing healthcare**^c^ (Yes vs. No)	0.47 (−0.22–1.16)	0.183		
**Currently on pain medication** (Yes vs. No)	0.40 (−0.13–0.92)	0.141		
**Cohort** (VIDUS vs. ACCESS)	0.02 (−0.51–0.55)	0.936		

^a^ Denotes activities/events within the week prior to the participant’s interview

^b^ Denotes activities/events within the month prior to the participant’s interview

^c^ Denotes activities/events within the 6 months prior to the participant’s interview

Table 3 presents the results of the bivariate and multivariate GLMs examining the effect of depressive symptoms on average functional pain interference. Here, moderate to severe depressive symptoms were found to be significantly associated with higher average pain interference scores in both the unadjusted (β = 1.49, 95% CI: 0.72–2.26, *p* < 0.001) and adjusted (β = 1.24, 95% CI: 0.33–2.15, *p* = 0.008) models. Based on the confounding model building approach, PTSD, anxiety, sex, ≥ daily heroin use, non-fatal overdose, and currently being prescribed pain medication were determined to be statistically significant positive confounders (based on the ≥5% change-in-estimate criterion) in the relationship between depressive symptoms and average pain interference. Of these, the final adjusted model showed statistical significance (based on *p*-value) for the effect of ≥ daily heroin use (β = 1.26, 95% CI: 0.47–2.05, *p* = 0.002), non-fatal overdose (β = 1.02, 95% CI: 0.08–1.96, *p* = 0.034), and being prescribed pain medication (β = 1.14, 95% CI: 0.48–1.81, *p* < 0.001) on average pain interference.

**Table 3:** Bivariate and multivariate generalized linear regression of the effect of depressive symptoms on average pain interference^a^ (*n* = 288)

	Unadjusted		Adjusted^**α**^	
Characteristic	β (95% CI)	***p -*** value	β (95% CI)	***p -*** value
**Depressive symptoms**^b^ (Moderate-Severe vs. Mild-None)	1.49 (0.72–2.26)	< 0.001	1.24 (0.33–2.15)	0.008
**PTSD**^**c**^ (Yes vs. No)	0.80 (0.11–1.49)	0.024	0.28 (−0.42–0.98)	0.437
**Anxiety**^b^ (Moderate-Severe vs. Mild-None)	0.94 (0.20–1.68)	0.013	0.13 (− 0.74–1.00)	0.762
**Age** (≥ Median vs. < Median)	−0.08 (− 0.78–0.61)	0.818		
**Sex** (Male vs. Female)	− 0.85 (− 1.58 - -0.13)	0.022	−0.59 (− 1.30–0.12)	0.106
**Race** (White vs. BIPOC or other)	0.00 (− 0.70–0.70)	0.999		
**Highest education completed** (≥ vs. < High school)	−0.20 (− 0.89–0.50)	0.581		
**Homeless**^**c**^ (Yes vs. No)	1.05 (0.03–2.06)	0.045		
**Incarcerated**^**c**^ (Yes vs. No)	−0.34 (− 2.07–1.40)	0.705		
**≥ Daily heroin use**^**c**^ (Yes vs. No)	1.53 (0.71–2.35)	< 0.001	1.26 (0.47–2.05)	0.002
**≥ Daily stimulant use**^c^ (Yes vs. No)	0.93 (0.23–1.63)	0.010		
**≥ Daily non-medical prescription opioid use**^c^ (Yes vs. No)	1.40 (−0.85–3.64)	0.225		
**≥ Daily cannabis use**^c^ (Yes vs. No)	−0.07 (− 0.87–0.73)	0.858		
**Heavy alcohol use**^**c**^ (Yes vs. No)	0.18 (−0.85–1.22)	0.730		
**Overdose**^**c**^ (Yes vs. No)	1.40 (0.42–2.38)	0.005	1.02 (0.08–1.96)	0.034
**Binge drug use**^**c**^ (Yes vs. No)	0.54 (−0.21–1.28)	0.158		
**Injection drug use**^**c**^ (Yes vs. No)	1.22 (0.53–1.92)	< 0.001		
**Inability to access addiction treatment**^c^ (Yes vs. No)	2.10 (−0.14–4.34)	0.067		
**Barriers to accessing healthcare**^c^ (Yes vs. No)	0.77 (−0.14–1.67)	0.098		
**Currently on pain medication** (Yes vs. No)	0.88 (0.20–1.57)	0.012	1.14 (0.48–1.81)	< 0.001
**Cohort** (VIDUS vs. ACCESS)	0.77 (0.08–1.46)	0.029		

^a^ Denotes activities/events within the 24 h prior to the participant’s interview

^b^ Denotes activities/events within the week prior to the participant’s interview

^c^ Denotes activities/events within the month prior to the participant’s interview

^c^ Denotes activities/events within the 6 months prior to the participant’s interview

^**α**^ In an additional sensitivity analysis excluding PTSD and anxiety in an adjusted model, the estimates for the remaining variables were: depressive symptoms (β = 1.46 [95% CI: 0.68–2.23], *p* < 0.001), ≥ daily stimulant use (β = 0.69 [95% CI: 0.01–1.37], *p* = 0.049), overdose (β = 1.08 [95% CI: 1.12–2.03], *p* = 0.028), currently on pain medication (β = 1.15 [95% CI: 0.48–1.81], *p* < 0.001)

In terms of potential differences between the two cohort samples, no significant differences were found for average pain severity (Table 2). Average pain interference was significantly greater among the VIDUS cohort in bivariable analysis (β = 0.77, 95% CI = 0.08–1.46, *p* = 0.029) but was not found to be statistically significant in the multivariable model building procedure (Table 3).

In both multivariate GLM models, no issues were identified related to multicollinearity between covariates (all VIF < 1.6). Regardless, a sensitivity analysis was performed excluding the covariates of PTSD (VIF = 1.15) and anxiety (VIF = 1.55) to assess any change in estimate when these covariates were excluded from the adjusted effect of depressive symptoms on functional pain interference (a sensitivity analysis was not performed on the pain severity model, as these covariates were not determined to be confounders in the adjusted model). The results yielded a higher estimate for the effect of depressive symptoms on pain interference (β = 1.46, 95% CI: 0.62–2.23, *p* < 0.001). The estimates for the remaining covariates remained similar to the original adjusted model, with the exceptions of ≥ daily stimulant use replacing ≥ daily heroin use as a significant confounder, and sex being excluded from the model as an insignificant confounder (Table 3, footnote).

## Discussion

This study of PWUD with major or persistent pain found that, in a sample of 288 participants among whom approximately one-quarter reported moderate to severe depressive symptoms, such depressive symptoms were significantly associated with greater average functional pain interference, but not significantly associated with average pain severity. Greater pain interference was also significantly associated with daily heroin use and non-fatal overdose, while other substance use factors such as daily prescription opioid or cannabis use did not appear to be significantly associated with average pain interference or severity.

The finding that depressive symptoms were significantly associated with greater pain-related functional interference, but not pain severity, is somewhat surprising and contrary to the hypothesis that both outcomes would be significantly associated with depressive symptoms. One explanation could be that perhaps in a substance-using population in particular, depression may have a greater impact on the pain-related functional activities of daily living than on the physical pain experience itself, although literature that points to the influence of depressed emotions and mood on pain perception supported our original hypothesis [41–43]. The significant effect of depressive symptoms on pain interference, as hypothesized, is supported by past research that has found that individuals with concurrent pain, substance use disorder, and mental health disorder have poorer functional outcomes in physical, personal, and social domains [29]. It is possible that there are overlapping functional impairments as a consequence of both pain and depressive symptoms, with regard to the domains quantified in this study using the BPI (i.e., general activity, mood, walking, relations with other people, sleep, and enjoyment of life). The relationship may even be cyclical in nature, as greater pain-related functional interference may lead to more distress and depressive symptoms, which in turn may lead to lower levels of function due to the inhibitive physical, mental, and social effects of the mental illness. The exact contribution of depressive symptoms and pain on functional interference is difficult to disentangle and likely varies across individual experiences. Research in this area supports the possibility that depressive symptoms may contribute to pain interference (e.g., decreased activity and mobility, decreased motivation or adherence toward treatment), or that depressive symptoms may be a consequence of pain interference (e.g., emotional distress, lower quality of life, greater psychosocial burden) [14–16].

The finding that non-fatal overdose was significantly associated with higher average pain interference is concerning, given that non-fatal overdose is a significant predictor of subsequent fatal overdose in PWUD [44]. In a study that examined characteristics of fatal opioid overdose decedents, the prevalence of mental illness and chronic pain were significantly higher in decedents with non-problematic opioid use—that is, in individuals without a diagnosis of opioid use disorder or evidence of aberrant drug-related behaviours within the year prior to death—compared to decedents with diagnosed problematic opioid use [5]. Greater attention to this less recognizable undiagnosed group, which constituted 53% of the deaths in the aforementioned study, is warranted given that their risk for overdose may not be as apparent as in individuals demonstrating problematic opioid use. In our models, non-fatal overdose was significantly associated with higher average pain interference, but not pain severity. Future research could explore potential pathways or mediating effects between pain-related functional limitations, depressive symptoms, and intentional or non-intentional overdose. Other studies have found greater likelihood of depression among those dying due to an unintentional opioid overdose [45] and a 26% (95% CI: 1–58%) increase in opioid-related deaths per 1% increase in state-level depression diagnoses [46]. One potential pathway may be the relationship between comorbid pain interference and depression on subsequent suicidal ideation and risk for overdose. Suicide has been deemed a “silent contributor” to the opioid overdose crisis: populations with chronic pain and mood disorders are more likely to receive opioid prescriptions, while also being at greater risk for suicide [47]. A study of opioid-using veterans found that greater pain severity, pain interference, depression, and suicidal ideation were positively related to greater overdose risk behaviours [31]. These findings may provide evidence that supports the concept of “adverse selection” currently being observed in treatment settings, in which individuals at high risk for poor outcomes are often those prescribed high-dose and high-risk chronic opioid treatment, in spite of clinical guidelines that recommend careful selection of low-risk patients for opioid treatment [48–50]. Collectively, these findings highlight the need for further research on and clinical assessment of precipitating factors for overdose among PWUD with pain, particularly those with comorbid depressive symptoms.

Regarding substance use related factors, we had hypothesized that greater pain severity and interference would be associated with greater intensity of substance use. Our multivariate models found that only daily heroin use remained significantly associated when analysed alongside the main effect of depressive symptoms on pain interference and controlling for other confounders including currently being prescribed pain medication. A possible explanation could be that heroin may be the substance of choice for this particular sample of respondents with pain, perhaps due to its rapid onset of effect compared to prescription opioids, or its lower price in unregulated drug markets compared to prescription opioids [51, 52]. It is interesting to note that heroin use was significantly associated with pain-related functional interference but not the severity of pain itself, which may suggest a correlation between heroin use and its effects on the functional domains of activity, mood, walking, relations with other people, and enjoyment of life, which may influence the consumption of the drug more than the goal of achieving analgesia for pain. These findings are also noteworthy in the context of opioid-induced hyperalgesia and withdrawal-associated injury site pain, which may contribute to an individual’s experience of pain and pain-related functioning [53]. These findings, along with literature indicating a resurgence of heroin use in recent years [54], signify a need for further research on the underlying reasons for heroin use among PWUD living with pain, as well as research on the temporal pathways through which PWUD with pain may initiate heroin use (e.g., whether an opioid use disorder preceded pain that may be result of opioid-induced hyperalgesia, or whether pain may have preceded opioid use that could have been precipitated iatrogenically via prescription opioids), the findings of which may lead to important insights for harm reduction and clinical management interventions. Contrary to our hypothesis, other substance use factors—such as daily prescription opioid misuse, daily cannabis use, or injection drug use—were not significantly associated with either pain severity or interference. Future studies may shed light on whether more complex interactions exist between depressive symptoms, pain, and other substance use behaviours, such as was found in one gender-based study that found lower levels of cannabis use were associated with higher levels of pain interference among females, but no such association was found among males [55].

There are several limitations of this study that should be mentioned. While the use of prescribed pain medication was accounted for in the analyses, we were unable to take into account any use of prescribed psychiatric medication that may affect the observed estimates. We can hypothesize that untreated depressive symptoms may have a greater effect on pain severity and interference, and that the inverse may also be true. The varying duration of recall periods for different measures in this study may also have affected the observed estimates. For instance, the BPI-SF measured past-week pain severity and past-24 h functional pain interference; the PROMIS measured past-week depressive symptoms; and the substance use variables were measured within a past-six months timeframe. This study also relied on self-reported data that may be subject to reporting bias including socially desirable responses. While efforts were made to use standardized, valid and reliable instruments as part of the interviewer-administered questionnaires, some of the measures used have not been tested for internal consistency. Additionally, as previously noted, the directionality of temporal associations cannot be established from the present analyses due to its cross-sectional nature, so the results should be interpreted with caution. These findings may not be generalizable to other groups of PWUD whose substance use, mental health, and pain characteristics may differ. The sample size (*n* = 288) for this study is relatively small, which may increase the margin of error of the reported estimates. Finally, data collection for this study occurred during a period of widespread fentanyl contamination in the study setting’s local drug supply [56], which may affect the estimates related to substance use (e.g., participants may have been using, knowingly or unknowingly, a substance with fentanyl added to it, which may increase the potential for misclassification bias in these analyses) and non-fatal overdose (i.e., a major increase in non-fatal overdose due to fentanyl contamination was observed during the study period, which may have increased the observed prevalence and correlates of overdose in these analyses).

In conclusion, this study has demonstrated that depressive symptoms appear to be significantly associated with greater functional pain interference, but does not appear to be significantly associated with average pain severity. Non-fatal overdose and daily heroin use were found to be significant confounders in the relationship between depressive symptoms and pain interference, and were independently associated with greater pain interference. These results highlight the importance of assessing for and addressing depressive symptoms, their related risk behaviours and harms such as heroin use and overdose, and the potential overlapping impacts with pain-related functional impairment, which have direct implications for wellbeing, quality of life, and treatment outcomes among PWUD with pain.

## Data Availability

The datasets used and/or analysed during the current study are not publicly available due to limitations of ethical approval involving participant data and anonymity, but may be made available from the corresponding author upon reasonable request.

## Abbreviations

ACCESS: AIDS Care Cohort to evaluate Exposure to Survival Services
BIPOC: Black, Indigenous, or Person of Color
BPI-SF: Brief Pain Inventory – Short Form
CDN: Canadian Dollar
CI: Confidence interval
DSM: Diagnostic and Statistical Manual of Mental Disorders
GLM: Generalized linear regression modelling
HIV: Human immunodeficiency virus
IQR: Interquartile range
PROMIS: Patient-Reported Outcomes Measurement Information System
PCL-5: Posttraumatic Stress Disorder Checklist for DSM-5
PTSD: Posttraumatic Stress Disorder
PWUD: People who use drugs
VIDUS: Vancouver Injection Drug Users Study
VIF: Variance inflation factor
